# Evaluating CloudResearch’s Approved Group as a solution for problematic data quality on MTurk

**DOI:** 10.3758/s13428-022-01999-x

**Published:** 2022-11-03

**Authors:** David J. Hauser, Aaron J. Moss, Cheskie Rosenzweig, Shalom N. Jaffe, Jonathan Robinson, Leib Litman

**Affiliations:** 1 Department of Psychology, Queen’s University, Kingston, ON Canada; 2 CloudResearch, Queens, NY USA; 3 Department of Clinical Psychology, Columbia University, New York, NY USA; 4 Department of Psychology, Lander College, Flushing, NY USA; 5 Department of Computer Science, Lander College, Flushing, NY USA

**Keywords:** Data quality, Test validity, Participant recruitment, Response bias

## Abstract

Maintaining data quality on Amazon Mechanical Turk (MTurk) has always been a concern for researchers. These concerns have grown recently due to the bot crisis of 2018 and observations that past safeguards of data quality (e.g., approval ratings of 95%) no longer work. To address data quality concerns, CloudResearch, a third-party website that interfaces with MTurk, has assessed ~165,000 MTurkers and categorized them into those that provide high- (~100,000, Approved) and low- (~65,000, Blocked) quality data. Here, we examined the predictive validity of CloudResearch’s vetting. In a pre-registered study, participants (*N* = 900) from the Approved and Blocked groups, along with a Standard MTurk sample (95% HIT acceptance ratio, 100+ completed HITs), completed an array of data-quality measures. Across several indices, Approved participants (i) identified the content of images more accurately, (ii) answered more reading comprehension questions correctly, (iii) responded to reversed coded items more consistently, (iv) passed a greater number of attention checks, (v) self-reported less cheating and actually left the survey window less often on easily Googleable questions, (vi) replicated classic psychology experimental effects more reliably, and (vii) answered AI-stumping questions more accurately than Blocked participants, who performed at chance on multiple outcomes. Data quality of the Standard sample was generally in between the Approved and Blocked groups. We discuss how MTurk’s Approval Rating system is no longer an effective data-quality control, and we discuss the advantages afforded by using the Approved group for scientific studies on MTurk.

## Introduction

For most of the past decade, Amazon Mechanical Turk (MTurk) has provided a reliable online source of participants for many experiments and surveys in the social sciences (Buhrmester et al., 2011, 2018; Paolacci et al., 2010; Litman & Robinson, 2020a). After gaining popularity around 2010, MTurk data represented nearly 50% of studies reported in top psychology journals by 2016 (Zhou & Fishbach, 2016) and by 2018 MTurk had been cited in more than 1000 different journals (Buhrmester et al., 2018). However, in 2018 significant issues with data quality emerged (e.g., Bai, 2018; Ryan, 2018; Chmielewski & Kucker, 2020). Despite solutions aimed at weeding out poor-quality respondents, concerns about data quality remain. In this paper, we validate a novel large-scale data quality solution that capitalizes on CloudResearch’s ability to vet the MTurk population and collate MTurker data from thousands of academic researchers.

### Data quality concerns, past and future

Data quality of MTurkers has often been a concern. Even though many researchers initially observed that data from MTurk was high quality (e.g., Buhrmester et al., 2011; Hauser & Schwarz, 2016), some studies also reported a troublingly high proportion of MTurkers providing poor-quality responses to surveys (Goodman et al., 2013, Necka et al., 2016). The MTurk “bot crisis” in 2018 exacerbated these concerns. Large proportions of MTurk respondents failed to notice reverse-coded items and responded to questions that required text responses with nonsense answers (Bai, 2018; Ryan, 2018). Furthermore, these poor responses appeared to originate from a few geolocations, which researchers interpreted as evidence that some enterprising MTurkers had created computer programs (i.e., “bots”) that would repeatedly “participate” in surveys to accrue incentives (e.g., Storozuk et al., 2020). Follow-up research identified “bot” responses as likely non-US respondents using virtual private networks (VPNs) to access surveys that were restricted to US MTurkers (Kennedy et al., 2020a, b; Litman et al., 2021).

Fortunately, there are many potential solutions to data quality issues. Instructional manipulation checks (IMCs) can identify inattentive participants (Oppenheimer et al., 2009). Online tools can block participants originating from suspicious geolocations (e.g., Gautam et al., 2018; Moss & Litman, 2018; Kennedy et al., 2020a, b). MTurk sample restrictions can target participants who have good “reputations” for providing valid data (Peer et al., 2014). While each has its drawbacks, these and other solutions have been leveraged to maximize data quality on MTurk in the past (Hauser et al., 2019; Chandler et al., 2020).

Unfortunately, these solutions are presently insufficient for ensuring high-quality data on MTurk. The reasons why are that (a) MTurkers learn from experience with IMCs and other attention checks that are often recycled and seldom updated (e.g., Hauser & Schwarz, 2016), (b) location-based tools are easily circumnavigated and not suited for identifying people at an individual level (e.g., Dennis et al., 2020), and (c) the current reputation-based system for identifying “high-quality” and “low-quality” respondents on MTurk is broken.

The inefficacy of the reputation system, in particular, is important because reputation has been widely used in the past to maintain data quality on MTurk (e.g., Peer et al., 2014). Once a participant signs up, their “reputation” is tracked through the proportion of surveys that they have had approved or rejected. The logic is that if a person provides poor-quality data, then researchers will reject their surveys. A history of rejected surveys will leave an MTurker with a low HIT acceptance ratio (HAR), meaning that the person would have a poor data quality reputation. Because researchers can require MTurkers to meet a certain HAR threshold for entry into their survey, the reputation system could, in theory, be used to ensure data quality (Peer et al., 2014).

While this system may have worked in the past, it is useless now. The reason for this is because very few social science researchers using MTurk reject HITs (human intelligence tasks), regardless of data quality. Out of the thousands of researchers using CloudResearch, for example, 49% have never rejected a single HIT and 38% have rejected less than 1% of participant submissions (Litman & Robinson, 2020b). Across 40 million HITs on CloudResearch, just 0.5% of survey responses have been rejected (Litman & Robinson, 2020b). It seems unreasonable to presume that only 0.5% of the surveys taken contain poor-quality data when the modal rate for poor-quality responses within the literature on data quality is between 8 and 12% (Curran, 2016). Thus, participants who provide poor-quality data have high reputations and are welcomed into surveys even when the required HAR is high.

Why don’t researchers reject more poor-quality surveys? There are several impediments. Many ethical review boards apply the same protocols to MTurk participants as they do to undergraduate participants, forbidding researchers from withholding incentives (which are contingent on survey approval). Additionally, assessing data quality takes effort and time (Hauser et al., 2019). There are no clear standards for evaluating quality, so researchers are burdened with wading through many measures to distinguish “acceptable” from “unacceptable” data. Finally, researchers have a responsibility to provide the incentives that are promised to participants. Rejecting surveys carries the risk of rejecting work that could possibly have been done by a person in good faith. Thus, rejections are rare.

### Data quality solutions for the future

So, how can one ensure high-quality data in a population where respondents learn how to pass data quality filters without necessarily providing high-quality data, where location-based tools are inadequate, and where the researchers do not (or cannot) use reputation tracking systems to identify high- vs. low-quality respondents? A large-scale solution that avoids the limitations of past measures and does not require researchers to reject participants who provide poor-quality data is needed.

CloudResearch’s Approved Group of participants attempts to do just that. CloudResearch is a third-party website that assists researchers with conducting studies on MTurk (Litman et al., 2017). Over the past 6 years, about 10,000 researchers have used CloudResearch to conduct over 340,000 MTurk studies with approximately 500,000 unique participants and over 50 million completed assignments. Thus, by virtue of its role as intermediary between social scientists and MTurk, CloudResearch is uniquely positioned to provide guidance regarding the data quality reputations of MTurkers.

The system CloudResearch has constructed relies on three types of information: (1) researcher generated data, (2) a series of open- and closed-ended instruments that are administered to MTurkers, and (3) technological measures such as geolocation tracking that are gathered by CloudResearch. Collectively, these measures are aimed at identifying a participant’s level of attention and capability to accurately respond to survey items. People who demonstrate that they are unwilling or unable to provide quality data are added to a Blocked List of participants.

The researcher-generated data CloudResearch relies on comes from the Universal Exclude List—a feature that was introduced in 2018 as a response to data quality problems (Moss & Litman, 2018). Rather than reject participants who provide bad data after a study (MTurk’s approach), CloudResearch enables researchers to place participants onto a Universal Exclude List. Participants who are placed onto a researcher’s Universal Exclude List are blocked from all that researcher’s future studies (without having any of their prior studies rejected). By examining which participants are repeatedly flagged across multiple researchers, CloudResearch can identify participants with a “reputation” for low-quality data without relying on researchers to officially reject low-quality submissions. This is one way CloudResearch gains insight into participant data quality.

Another way CloudResearch vets participants is by administering surveys that contain data quality instruments to large swaths of MTurkers. The surveys CloudResearch administers pull from a large library of data quality measures that were designed to detect various forms of problematic responding and to ensure that participants do not see repeated stimuli (Litman et al., 2020).

Finally, the third pillar in CloudResearch’s vetting includes technical measures to assess whether participants are (a) accessing US-based studies from other countries, (b) using auto-fill plug-ins for matrix-type questions, (c) using the same device to attempt the survey more than once, and (d) routing web traffic through suspicious locations. By examining this data in conjunction with behavioral data and aggregating across thousands of external studies conducted by academics, CloudResearch can assess the data quality of MTurkers over time, across studies, and across academic labs. Since 2020, these measures have been used to vet 165,000 MTurkers. Vetted participants are classified into either a Blocked Group or an Approved Group. Our goal in this paper was to test the effectiveness of using the Approved Group for ensuring data quality.

## Current research

We investigated the predictive validity of CloudResearch’s Approved and Blocked Groups on data quality, examining whether Approved (vs. Blocked) status predicts higher-quality data on an array of measures. Blocked and Approved MTurkers were recruited for a study. As an additional comparison group, an “Open Sample” was gathered with conventional MTurk restrictions (i.e., 95% HAR and 100 approved HITs, U.S. location) but otherwise was open to all MTurkers. This group serves as an analogue for the data quality a researcher should expect when posting a survey to MTurk that uses standard data quality assurances from the past but does not use the CloudResearch filters for Approved and Blocked statuses. All participants completed measures assessing major data-quality concerns on the platform (for a review, see Hauser et al., 2019). We hypothesized that participants from the Approved Group would score higher on various indices of data quality than participants from the Blocked Group or the Open Sample. We also expected Open Sample participants to fall somewhere in between the other groups.

We report all studies, manipulations, measures, and exclusions. The data and materials for all experiments are available at: https://osf.io/7bznv/ and the study was preregistered: https://osf.io/xn2ed.

## Method

### Participants

Using CloudResearch’s MTurk Toolkit (Litman et al., 2017), we created four identical MTurk surveys that each invited different participants. The first survey recruited MTurkers in the CloudResearch Approved Group; the second recruited MTurkers in the CloudResearch Blocked Group; the third was open to all U.S. MTurkers with at least 100 completed HITs and a HAR 95% or higher (Hauser et al., 2019); and the fourth survey was open to all U.S. MTurkers with at least 1000 HITs completed and a HAR of 99% or higher (an “accelerated qualifications” group). All surveys were programmed into a “survey group” to ensure participants could only participate in one study. All participants were paid $1.25 and we expected the survey to take 12 min. We invited participants to the study with e-mail invitations and closed each survey when it reached our quota of 300 people. Sensitivity power analyses (Faul et al., 2007) indicate that this sample size provides 80% power for detecting effects between groups with effect sizes of *d* = 0.23, which is smaller than the effect sizes of most comparisons of attentiveness (Hauser & Schwarz, 2016). These sample sizes are also roughly in line with other studies that have investigated differences in effect sizes across research platforms (e.g., Peer et al., 2017).

Upon completion of the study, inspection of participant characteristics revealed that (a) over 95% of participants in the “accelerated qualifications” group were already vetted by CloudResearch and were thus already in either in the Approved or Blocked Groups, and (b) there were less than 3500 MTurkers in this group across the entire MTurk platform. Because this sample contained over 95% overlap with the other samples (Blocked and Approved) and because the small number of overall MTurkers who belong to this group appear to make it unfeasible for research at scale, we did not interpret the data of this group. The Standard sample was open to the entire MTurk pool and includes a substantial number of respondents who were not vetted by CloudResearch and are thus not in either the Approved or Blocked groups.

### Materials and procedure

Participants were directed to a survey in Qualtrics and responded to items assessing data quality.

#### Satisficing

Because participants sometimes skim text rather than read carefully (Krosnick, 1991), we presented two reading comprehension tasks that asked participants to read an article and answer three questions about it (Kane et al., 2020). Participants also responded to four attention checks with factually incorrect answers embedded within other scales (e.g., “I work 28 hours in a typical workday”).

They completed a replication of a study with a minor between-subjects difference in wording of three words within a 96-word vignette. Participants reported how much they would be willing to pay for a soda from a run-down grocery store vs. a fancy resort (randomly assigned). Attentive participants are typically willing to pay more at the fancy resort than the run-down grocery store (Oppenheimer et al., 2009).

Participants also completed the Big Five Inventory (BFI; John et al., 2008). To BFI items, we added ten direct antonyms. For example, “tends to be organized” was reversed and added as “tends to be disorganized.” These synonym–antonym pairs allowed us to examine individual-level reliability using the Squared Discrepancy Procedure (SDS; see Litman et al., 2015).

#### International respondents

One of the largest sources of low-quality data on MTurk comes from respondents outside of the U.S. who fraudulently access studies that are open to only US-based respondents (Moss et al., 2021; Kennedy et al., 2020a, b). Such participants often provide unconventional responses to text-response questions, occasionally answering with Google search results (Litman et al., 2021). To detect this behavior, participants identified the content of three images with text responses. We deliberately selected images for which reverse Google image searching would yield incorrect results.

Participants also responded to three Winograd schema questions (Weston et al., 2015; Levesque et al., 2012). These questions have been proposed as a type of a Turing test, requiring people to identify the antecedent of an ambiguous pronoun and to rely on commonsense reasoning. They are fairly simple for humans but present difficulties to computers and search engines. For instance, one item read “John is either in the classroom or the playground. Sandra is in the garden. Is John in the classroom? Yes, No, Maybe.”

We also asked: “Does the moon ever need a haircut?” with an open textbox response. Googling this question returns information about how to cut one’s hair based on their horoscope.

#### Replication of classic effects

##### Anchoring 1: Population of Chicago

Participants estimated the population of Chicago after being randomly assigned to an anchoring condition (between subjects). In the low (high) anchor condition, participants were asked whether the population of Chicago is more or less than 200,000 (5,000,000). People exposed to the high anchor tend to provide larger estimates than people exposed to the low anchor (Jacowitz & Kahneman, 1995).

##### Anchoring 2: Multiplication

Participants estimated the product of a series of numbers. The order of the numbers was randomly assigned (between subjects). In the descending (ascending) group, participants estimated the product of 8✕7✕6✕5✕4✕3✕2✕1 (1✕2✕3✕4✕5✕6✕7✕8). People provide larger estimates when exposed to the descending than ascending problem (Tversky & Kahneman, 1973).

##### Trolley dilemma

Participants were randomly assigned (between subjects) to different versions of the trolley dilemma. Participants were asked whether they would kill one person to save five by pulling a lever to turn the trolley onto another track (vs. by pushing a bystander in front of the train). People are typically more willing to sacrifice one life to save five when pulling the lever rather than pushing a person onto the tracks (e.g., Hauser et al., 2007).

#### Cheating

Online participants sometimes “cheat” by Googling questions when researchers explicitly ask them not to. We asked participants six questions about political and government facts (adapted from Clifford & Jerit, 2016) and asked them to forgo utilizing search engines. Afterward, participants self-reported whether they Googled answers.

TaskMaster recorded whether participants left the survey window (Permut et al., 2019), although there are other ways to detect cheating on these types of questions (see Motta et al., 2017; Smith et al., 2020). While participants could have left the survey to do things besides Googling the answers, we presume that throughout most of the survey this is random (not systematic) noise. Yet, when participants were asked political knowledge questions and explicitly asked not to Google the answers, we used this data as a proxy for cheating.

## Results

Table 1 contains overall means, standard deviations, and correlations among all variables except for the experimental manipulations.

**Table 1:** Means, standard deviations, and correlations

	M	SD	1	2	3	4	5	6	7
1. Mock Vignette	4.51	1.82	-	.669**	.558**	.601**	– .310**	.593**	– 0.79*
2. Attention Checks	3.45	1.04		-	.588**	.690**	– .409**	.642**	– .055
3. Squared Discrepancy Scores	3.51	1.42			-	.627**	– .410**	.585**	– .212**
4. Image Items Passed	2.35	0.98				-	– .500**	.616**	– .249**
5. Googled Items	0.14	0.41					-	– .394**	.154**
6. Winograd Performance	2.26	0.98						-	– .126**
7. Left Page (frequency)	0.27	0.44							-

* *p* < .05, ** *p* < .01

### Satisficing

#### Reading comprehension

The groups differed in how many reading comprehension questions they correctly answered, *F*(2, 852) = 71.35, *p* < 0.001, ${\eta }_{p}^{2}$ = .143. The Blocked Group (*M* = 3.57, *SD* = 2.06) answered fewer questions correctly than the Approved Group (*M* = 5.25, *SD* = 1.23), *t*(566) = – 11.96, *p* < 0.001, *d* = – 1.01, 95% CI [– 0.830, – 1.180], or the Open Sample (*M* = 4.61, *SD* = 1.71), *t*(553) = – 6.51, *p* < .001, *d* = – 0.55, 95% CI [0.383, 0.722]. Finally, the Open Sample answered fewer questions correctly than the Approved Group, *t*(585) = – 5.23, *p* < 0.001, *d* = – 0.43, 95% CI [0.268, 0.595].

#### Attention checks

The groups also differed in how many attention check questions they passed, *F*(2, 851) = 52.07, *p* < 0.001, ${\eta }_{p}^{2}$ = .109. The Blocked Group (*M* = 3.02, *SD* = 1.24) passed fewer checks than the Approved Group (*M* = 3.87, *SD* = 0.52), *t*(566) = – 10.77, *p* < .001, *d* = – 0.91, 95% CI [– 0.732, – 1.078], or the Open Sample (*M* = 3.42, *SD* = 1.09), *t*(552) = – 4.02, *p* < .001, *d* = – 0.34, 95% CI [– 0.173, – 0.509]. The Open Sample passed fewer checks than the Approved Group, *t*(584) = – 6.41, *p* < .001, *d* = – 0.53, 95% CI [– 0.365, – 0.694].

#### Soda Task

Although not pre-registered, we were surprised by the range of responses we received on questions with open-text responses, so we explored implausible responses in this and other open-response tasks as an aspect of data quality. In the soda task, participants reported being willing to pay between $0 and $780,000 for a soda, a range that clearly indicates data quality issues. We identified outliers (> $20) from boxplots (Tukey, 1977) and compared implausible responses across groups. Approved Group participants gave fewer implausible responses [3.0%] than the Open Sample [18.8%, χ^2^(1, *N* = 587) = 38.29, *p* < .001, $$\varphi$$ = – .255] or the Blocked Group [35.8%, χ^2^(1, *N* = 568) = 101.19, *p* < .001, $$\varphi$$ = – .422]. To reduce the impacts of outliers and unequal variances across conditions, we rank-transformed people’s willingness to pay (higher scores indicating a willingness to pay more) and conducted a 3 (sample: Approved, Open, Blocked) × 2 (store location: fancy resort, run-down grocery store) between-subjects ANOVA on ranked WTP.

**Fig. 1:**
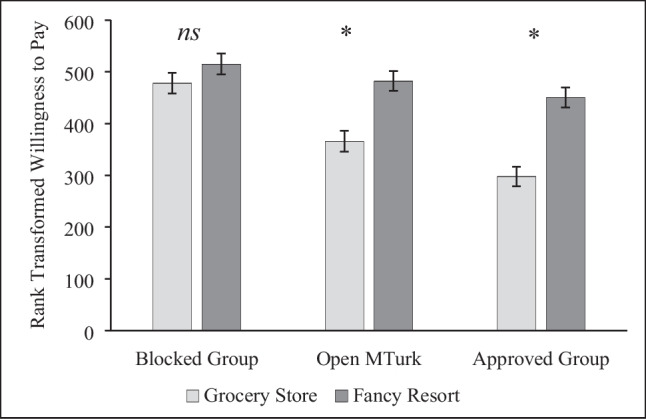
Store Location Effect in the Soda Task. Responses were rank transformed to minimize the impact of implausible answers. Higher numbers indicate a willingness to pay more for the soda. * indicates a significant difference at *p* < .001. Error bars show standard errors

The effect of the soda manipulation varied across groups, *F*(2, 845) = 4.44, *p* = .012, ${\eta }_{p}^{2}$ = .010 (Fig. 1). Store location had no impact on Blocked Group participants, *F*(1, 845) = 1.66, *p* = .198. This means that the framing effect in the soda task could not be replicated with the Blocked Group even after implausible scores were rank-transformed. By contrast, the manipulation had the expected effect on participants in the Open Sample, *F*(1, 845) = 17.53, *p* < .001, ${\eta }_{p}^{2}$ = .020, 90% CI [.008, .039], and a noticeably larger effect on the Approved Group, *F*(1, 845) = 31.67, *p* < .001, ${\eta }_{p}^{2}$ = .036, 90% CI [.018, .059].

#### Big Five Inventory

The Blocked Group produced lower reliability scores for all BFI subscales than did the Approved Group or Open Sample (see Table 2).

**Table 2:** Reliability Coefficients for the BFI

	Blocked Group	Open Sample	Approved Group
Openness	.76	.79	.85
Conscientiousness	.82	.85	.89
Extraversion	.69	.83	.89
Agreeableness	.76	.77	.85
Neuroticism	.79	.85	.92

**Fig. 2:**
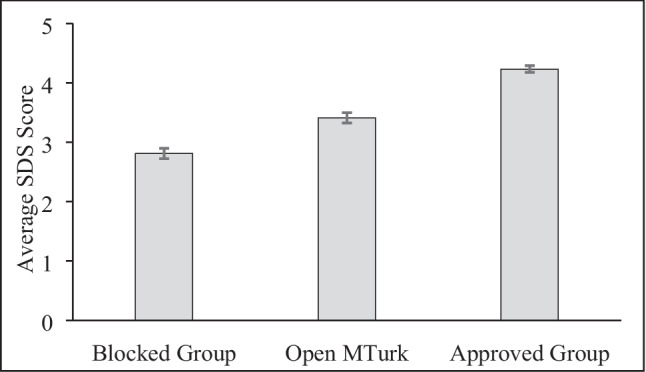
Squared Discrepancy Scores by Group. Z-transformed squared discrepancy scores range from 0 to 5 with higher scores indicating greater response consistency. Error bars show standard errors.

Likewise, there were group differences in BFI Squared Discrepancy Scores (Fig. 2), *F*(2, 852) = 86.21, *p* < .001, ${\eta }_{p}^{2}$ = .168. The Blocked Group had the lowest SDS. Both the Open Sample, *t*(553) = 4.98, *p* < .001, *d* = 0.42, 95% CI [0.591, 0.255], and the Approved Group, *t*(566) = 13.92, *p* < .001, *d* = 1.22, 95% CI [0.991, 1.347], had significantly higher scores. Finally, the Approved Group had significantly higher scores than the Open Sample, *t*(585) = 8.03, *p* < .001, *d* = 0.66, 95% CI [0.497, 0.829].

### International respondents

#### Image Identification

Coders identified whether image descriptions were in any way descriptive of the image and whether responses contained content from a reverse Google image search. The interrater reliability among coders was good; Cohen’s kappas .61 to 1.00. Disagreements were resolved by discussion.

The groups differed in the accuracy of their English language text responses to simple image identification tasks, *F*(2, 851) = 86.63, *p* < .001, ${\eta }_{p}^{2}$ = .169, and in the number of responses that showed evidence of being Googled, *F*(2, 851) = 20.64, *p* < .001, ${\eta }_{p}^{2}$ = .046.

**Fig. 3:**
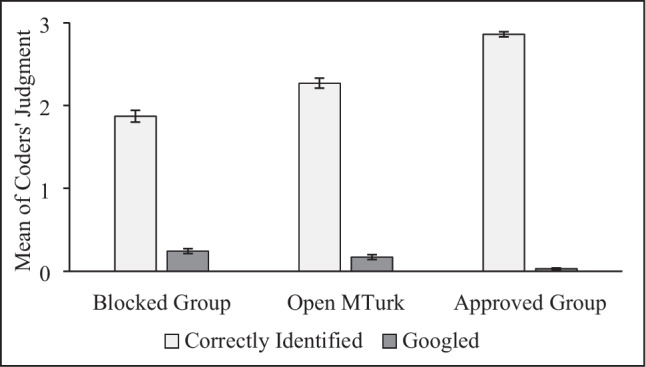
Coders’ Judgments for the Image Identification Task. Coders judged whether participants accurately identified the content of three simple images and whether participants appeared to Google the answer. Error bars show standard errors

The Blocked Group provided the least accurate responses and the most evidence of using Google. Specifically, the Blocked Group was less accurate than both the Open Sample, *t*(552) = – 4.38, *p* < .001, *d* = – 0.37, 95% CI [– 0.540, – 0.204], and the Approved Group, *t*(566) = – 14.42, *p* < .001, *d* = – 1.21, 95% CI [– 1.033, – 1.391] (Fig. 3). The Open Sample, in turn, was less accurate than the Approved Group, *t*(584) = – 8.87, *p* < .001, *d* = – 0.73, 95% CI [– 0.900, – 0.565].

When examining Googled responses, the Blocked Group showed more evidence of Googling than either the Open Sample, *t*(552) = 1.65, *p* = .05, *d* = 0.14, 95% CI [– .026, .307], or the Approved Group, *t*(566) = 6.96, *p* < .001, *d* = 0.59, 95% CI [0.416, 0.753]. Meanwhile, the Open Sample Googled more than the Approved Group, *t*(584) = 4.84, *p* < .001, *d* = 0.40, 95% CI [0.236, 0.563].

#### Winograd

The groups varied in performance on Winograd items, *F*(2, 852) = 56.72, *p* < .001, ${\eta }_{p}^{2}$ = .117. The Blocked Group (*M =* 1.83, *SD =* 1.11) answered fewer questions correctly than the Open Sample (*M =* 2.26, *SD =* 0.96), *t*(553) = – 4.94, *p* < .001, *d* = – 0.42, 95% CI [– 0.587, – 0.251], or the Approved Group (*M =* 2.65, *SD =* 0.65), *t*(566) = – 10.91, *p* < .001, *d* = – 0.92, 95% CI [– 1.090, – .0.744]. Likewise, the Open Sample answered fewer items correctly than the Approved Group, *t*(585) = – 5.75, *p* < .001, *d* = – 0.48, 95% CI [– 0.639, – 0.311].

#### Moon Haircut

Coders had high agreement (Cohen’s Kappa = .993) and resolved discrepancies by discussion. While almost everyone passed the moon haircut question in the Approved Group [97.7%] fewer did so in the Open Sample [91.6%, χ^2^(1, *N* = 587) = 10.66, *p* < .001, $\varphi$ = .135] and Blocked Group [83.6%, χ^2^(1, *N* = 555) = 8.37, *p* = .004, $\varphi$ = .123].

### Replication of classic effects

#### Anchoring 1: Population of Chicago

Answers ranged from “1.2” to “50 billion,” a range that again indicated data quality issues. For this item, however, all groups gave similar proportions of implausible responses, defined as answers > 9.5 million (Tukey, 1977), χ^2^(2, *N* = 854) = 2.76, *p* = .251. Percentages of implausible responses ranged from 5.2% to 8.7% across groups.

**Fig. 4:**
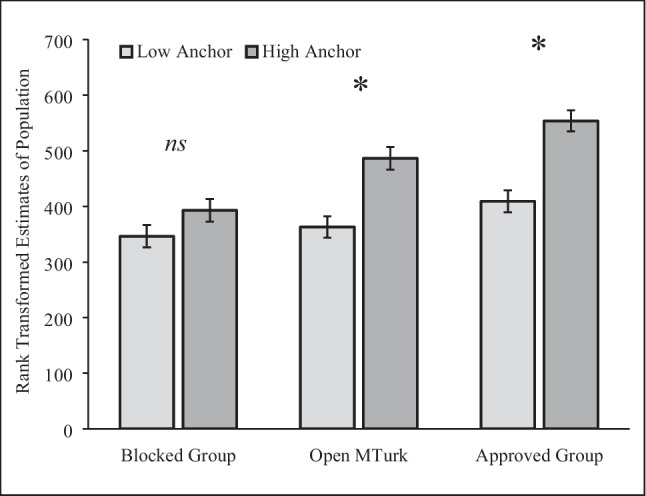
Rank Transformed Estimates for the Population of Chicago. Responses were rank transformed to minimize the impact of implausible answers. Higher numbers indicate higher population estimates. * indicates a significant difference at *p* < .001. Error bars show standard errors

The effect of the anchoring task varied across groups. A 3 (sample: Approved, Open, Blocked) × 2 (anchor: low, high) between-subjects ANOVA on rank transformed population estimates revealed a significant interaction between sample and anchor, *F*(2, 848) = 3.14, *p* = .04, ${\eta }_{p}^{2}$ = .007 (see Fig. 4). Anchoring had no effect on the Blocked Group, *F*(1, 848) = 2.52, *p* = .113. However, the typical anchoring effect replicated in the Open Sample, *F*(1, 848) = 19.29, *p* < .001, ${\eta }_{p}^{2}$ = .022, 90% CI [.009, .041], and the Approved Group, *F*(1, 848) = 26.29, *p* < .001, ${\eta }_{p}^{2}$ = .030, 90% CI [.001, .050].

#### Anchoring 2: Multiplication

Answers ranged from “0” to over “151 billion,” again revealing data quality issues. In addition, many participants provided the exact correct answer (40,320). When flagging outliers (> 99,616; Tukey, 1977) and correct answers, implausible responses were significantly more common in the Blocked Group (28%) than the Approved Group [11.4%, χ^2^(1, *N* = 567) = 25.12, *p* < .001, = – .210], and in the Open Sample (22.0%) compared to the Approved Group, [χ^2^(1, *N* = 586) = 11.87, *p* < .001, $\varphi$ = – .142]. We ranked transformed responses and conducted a 3 (sample: Approved, Open, Blocked) × 2 (anchor: ascending, descending) between-subjects ANOVA.

**Fig. 5:**
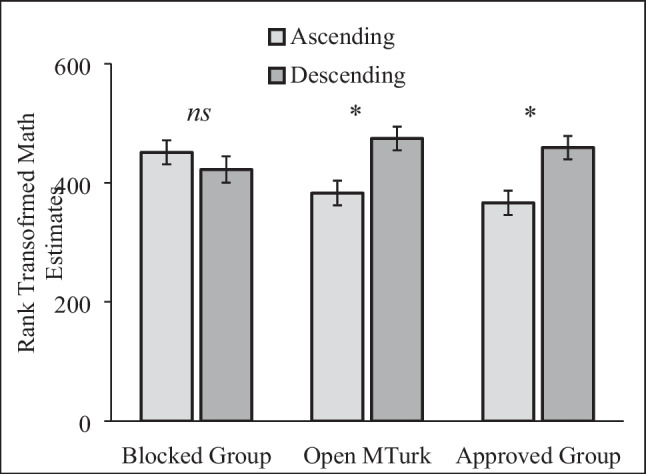
Rank Transformed Estimates for the Math Problem. Responses were rank transformed to minimize the impact of implausible answers. Higher numbers indicate higher product estimates. * indicates a significant difference at *p* < .01. Error bars show standard errors

The effect of the low vs. high anchor varied across groups, *F*(2, 848) = 5.54, *p* = .004, ${\eta }_{p}^{2}$ = .013, for the interaction of anchor and sample, (see Fig. 5). Anchoring had no effect on the Blocked Group, *F <* 1. By contrast, the typical anchoring effect replicated in the Open Sample, *F*(1, 848) = 10.01, *p* = .002, ${\eta }_{p}^{2}$ = .012, 90% CI [.003, .027], and Approved Group, *F*(1, 848) = 10.81, *p* = .001, ${\eta }_{p}^{2}$ = .013, 90% CI [.003, .028]. Thus, even after rank transforming responses, anchoring effects did not replicate on Blocked Group participants.

#### Trolley Dilemma

We conducted a 3 (sample: Approved, Open, Blocked) × 2 (anchor: ascending, descending) between-subjects ANOVA on trolley decisions (0 = stop; 1 = do not stop)^1^. The effect of trolley condition varied by group, *F*(2, 848) = 17.26, *p* < .001, ${\eta }_{p}^{2}$ = .039. The trolley effect replicated with a small effect size in the Blocked group, *F*(1, 848) = 17.62, *p* < .001, a moderate effect size in the Open Sample, *F*(1, 848) = 73.87, *p* < .001 and a large effect size in the Approved Group *F*(1, 848) = 168.74, *p* < .001 (Table 3).

**Table 3:** The percentage of people choosing to turn the trolley across conditions

	Classic		Footbridge		Difference	${\eta }_{p}^{2}$	90% CI
	*M*	*SD*	*M*	*SD*			
Blocked Group	1.15	0.36	1.36	0.48	.21	.020	[.008, .039]
Open Sample	1.14	0.35	1.57	0.50	.43	.080	[.053, .110]
Approved Group	1.16	0.37	1.78	0.41	.62	.166	[.130, 203]

Scores closer to one indicate a decision to not stop the trolley. Difference = the difference between the mean of the footbridge and classic version for each sample

### Cheating

#### Political Knowledge Questions

The groups differed in self-reported Googling of political knowledge questions, χ^2^(2, *N* = 855) = 60.13, *p* < .001, $\varphi$ = .265. The Blocked Group self-reported more cheating (26.12%) than either the Open Sample (14.63%), χ^2^(1, *N* = 555) = 23.20, *p* < .001, φ = .199, or the Approved Group (3.33%), χ^2^(1, *N* = 568) = 60.73, *p* < .001, $\varphi$ = .327. The Open Sample self-reported more cheating than the Approved Group, χ^2^(1, *N* = 587) = 23.20, *p* < .001, $\varphi$ = .199.

TaskMaster metadata confirmed self-reported cheating trends. There were group differences in how often participants left the survey window, χ^2^(2, *N* = 855) = 17.31, *p* < .001 $\varphi$ = .142. While 33.2% of people in the Blocked Group left the window during political knowledge questions, 18.3% of people in the Approved Group did so, χ^2^(1, *N* = 587) = .914, *p* < .001. The Open Sample was not significantly different from the Blocked Group with 28.9% of people leaving the survey window, but was significantly higher than the Approved Group, χ^2^(1, *N* = 587) = 9.14, *p* = .002.

## General discussion

Are CloudResearch’s Approved and Blocked groups valid predictors of data quality in social science surveys? Our results suggest they are. Participants in the Approved Group, compared to those in the Blocked Group and the standard Open MTurk sample, i) identify the content of images more accurately, ii) accurately answer more reading comprehension questions, iii) respond to reversed coded items more consistently, iv) pass a greater number of attention checks, v) self-report less cheating and actually leave the survey window less often on easily-Googleable questions, vi) replicate classic psychology experimental effects more reliably, and vii) answer AI-stumping questions more accurately. Many of the common data quality concerns that researchers hold about the MTurk participant pool, such as satisficing, non-native language speakers, misrepresentation, and cheating (Hauser et al., 2019), are mitigated by the Approved Group. While the Open Sample often produced data quality somewhere between the Blocked and Approved Groups, there were still more indications of inattention, random responding, implausible open-ended answers, and other data quality issues in the Open Sample than the Approved Group. Often, these data quality issues translated into “nosier” data with smaller effect sizes. In the Discussion below, we outline the implications these various groups of MTurkers have for replicability of experimental effects and methods of maintaining data quality.

### Implications

#### Collecting standard MTurk samples is not sufficient for replicating prior MTurk studies

How researchers sample from MTurk has implications for replicating past effects. For instance, recent large-scale collaborative projects have proposed to examine whether findings from MTurk studies conducted between 2015 and 2018 replicate on current MTurk samples (Mechanical Turk Replication Project, 2021). One criterion for conducting faithful replications is to consider and account for conceptual differences between the original research and the replication attempt (Brandt et al., 2014; Ramscar, 2016; Schwarz & Strack, 2014). Even though a new project may exactly replicate the procedures of prior studies, the effect may not replicate when procedures are no longer sufficient for manipulating the same conceptual constructs as before (see Luttrell et al., 2017, for an example of when construct validation requires that new procedures are necessary to replicate old conditions).

The same is true for recruitment criteria. Replicating pre-2018 MTurk recruitment criteria in current MTurk studies **is** no longer sufficient for gathering samples of comparable quality (Chmielewski & Kucker, 2020; Bai, 2018; Kennedy et al., 2020a, b). Since 2018, researchers have noted an increase in the number of international workers on MTurk gaining access to studies meant for people in the U.S. (Kennedy et al., 2020a, b; Litman et al., 2021). When combined with more mundane data quality issues among U.S. participants (e.g., satisficing, inattention), more than one-third of people may provide low quality data (as CloudResearch’s overall vetting suggests). The results from the present study demonstrate the effect these participants can have within studies. Across most measures in our study, poor data quality led to either an attenuation of effect sizes (Open Sample) or a failure to replicate some of the most robust and well-documented effects within psychology (Blocked Group).

Because there are well-documented trends with data quality on MTurk, good faith replications of past findings should ensure that MTurk samples are of comparable data quality to the original research’s samples. The CloudResearch Approved Group may be a way to do this. As the current findings demonstrate, CloudResearch Approved participants have higher data quality than the typical MTurk samples which have declined in quality since 2018 (i.e., U.S. participants with 95% HAR). Furthermore, CloudResearch uses a uniform set of measures to vet research participants. When researchers gather data from MTurk, they are forced to make idiosyncratic decisions for how to detect and remove problematic participants. A failure to detect these participants adds noise to a dataset and differences in the measures researchers choose to assess data quality adds systematic variability to replication attempts. Thus, utilizing CloudResearch Approved participants may be a suitable measure for ensuring comparability of data quality in MTurk samples across research labs.

#### HIT acceptance ratio is not a sufficient condition for data quality on MTurk

These results imply that one widespread belief about MTurk needs to be updated: that of reputation, as measured by HAR, being sufficient for maintaining data quality (Peer et al., 2014). Unfortunately, this is no longer be true. On most measures of data quality, Open Sample participants performed poorly relative to historical benchmarks. While MTurkers with 95% HAR or above used to pass all attention checks in a study at rates between 80% and 90% (Peer et al., 2014), only 72% passed all checks in our study. Further, only about 60% of the Open Sample correctly explained the content of three very simple image identification questions, and we flagged approximately one-fifth of their answers on anchoring tasks because they were implausible. The standard MTurk-based qualifications do not work anymore presumably because researchers do not reject HITs containing poor-quality data. Almost 90% of researchers using MTurk reject less than 1% of participant submissions, and nearly half never reject any submissions (Litman & Robinson, 2020b). Either a critical mass of researchers must reject poor-quality HITs or a different system (such as Approved workers) is needed. Given that the current reputation system amounts to a collective action problem, there is little reason to expect researchers will begin rejecting poor-quality HITs (see Ahler et al., 2019).

### Additional considerations

The current results, and prior studies assessing data quality on MTurk, should be considered as a “snapshot in time”. Data quality on MTurk, along with best practices for conducting research on the platform, constantly change. MTurkers learn, and some never leave the platform, so simple tricks that once maximized data quality (e.g., U.S. country of residence restrictions) are often countered (e.g., utilizing VPNs to spoof U.S. locations). While the Approved Group goes a long way to providing quality data, survey-level design considerations to maximize data quality should also be implemented. Surveys should be no longer or more tedious than necessary (Hauser et al., 2019). Heaven forbid, they could even be fun. It would be unreasonable to expect high-quality data when under-incentivizing and overtaxing participants. Hence, using something like CloudResearch’s Approved Group is not the only factor that affects data quality.

  If researchers heed these precautions, it should be possible to gather quality data from MTurk without sacrificing the demographic composition of participants. As shown in Table 4, the demographics of people in the Approved Group match those of the MTurk population quite well in terms of age, gender, race, ethnicity, education, and income. For instance, females make up 60% of the Approved group (vs. 54% of standard MTurk), 46% of the Approved group are Democrats (vs. 45% of standard MTurk), 34% of the Approved group has a bachelor’s degree (vs. 38% of standard MTurk), and 8% of the Approved group reports an annual household income in the $60,000–70,000 bracket (vs. 8% of standard MTurk). There do not appear to be large representativeness differences between the Approved group and a sample collected via standard MTurk recruitment criteria.

**Table 4:** Basic demographics of Approved Group participants and the MTurk population

	Approved Group	Standard MTurk
Age		
18–29	33.3	30.8
30–39	36.1	37.3
40–49	16.9	17.6
50–59	9.0	9.2
60–69	3.9	4.1
70+	0.9	0.9
Gender		
Male	39.7	45.7
Female	60.3	54.3
Political party		
Democrat	46.2	44.7
Republican	25.8	29.2
Other	28.0	26.0
Race		
White	76.8	72.0
Black	9.1	11.8
American Indian or Alaska Native	0.8	1.7
Asian	7.4	9.7
Native Hawaiian or Pacific Islander	0.2	0.2
Other	5.6	4.6
Hispanic		
Yes	10.3	15.2
Highest degree		
No college degree	40.8	36.2
Associate degree	11.3	9.9
Bachelor’s degree	33.7	37.9
Graduate degree	14.2	16.1
Household income		
< $10,000	5.5	5.8
$10,000–$19,999	6.6	7.1
$20,000–$29,999	10.6	10.8
$30,000–$39,999	11.6	11.5
$40,000–$49,999	10.1	10.6
$50,000–$59,999	10.2	11.4
$60,000–$69,999	7.6	7.7
$70,000–$79,999	8.3	8.3
$80,000–$89,999	5.1	4.8
$90,000–$99,999	5.3	5.0
$100,000–$149,000	13	11.6
>$150k	6	5.2

Even though the Approved Group consists of tens of thousands of active MTurkers, some researchers may wonder whether it is large enough to avoid sensitization effects or the problems that come from exposing the same set of participants to the same measures too often. We believe this concern is no greater with the Approved Group than any other commonly used source of online participants for three reasons. First, CloudResearch’s vetting appears to primarily remove inattentive and fraudulent participants from outside of the U.S. (Litman et al., 2021), which is a group outside of most researchers’ target population. Second, the Approved Group is continuously growing; each month several thousand new accounts are added. Finally, researchers can impose a maximum number of HITs completed criteria when sampling from MTurk, effectively capping participant experience within the sample (see Robinson et al., 2019). Together, these factors, suggest that non-naivete should not be worse with the Approved Group than other online sources.

Beyond ensuring data quality, CloudResearch’s Approved/Blocked lists have the benefit of increasing standardization in measurement. When researchers add attention checks, red herrings, and other measures meant to assure quality to their studies, they often select measures that appear face valid but are of questionable psychometric qualities (e.g., Berinsky et al., 2014). Furthermore, researchers vary greatly in the standards they set for judging quality and deciding which participants to exclude or retain from analyses. When not evaluated in good faith (e.g., throwing out data from participants who disconfirm the hypothesis), this can constitute questionable research practices. Perhaps most critically, research papers often fail to adequately describe the details about how participants were sampled, screened, and evaluated in terms of quality. The cumulative effect of these decisions is an unknown amount of variability between researchers and labs that may contribute to a replication’s failure or success. In contrast to this variable approach, CloudResearch’s vetting offers a standardized procedure for assessing quality. The data reported in this paper provide a benchmark for population estimates of problematic participants (~30%) and validate the procedures used to vet participants.

In conclusion, CloudResearch’s Approved Group appears to be one way to overcome issues with data quality on MTurk. The Approved Group may succeed where other methods fail because it does not rely on researcher rejections or repeatedly measuring attention with the same items. Because CloudResearch can aggregate participant data across thousands of academic users to establish an independent data-quality filter, it has the potential to evolve as flexibly as bad actors do and remain viable into the future.

## Data Availability

The data, materials, analysis code, and preregistrations are available in the OSF repository at: https://osf.io/7bznv/.

## Footnotes

1 We analyzed this with ANOVA for ease of interpretation and consistency with prior tests (Gomila, 2020), but note that binary logistic regression yields similar conclusions.

## Declarations

### Open Practices Statement

The data and materials for all studies are available at https://osf.io/5zdwy/. The study was preregistered https://osf.io/xn2ed.
